# Changes in Protein Expression in Warmed Human Lens Epithelium Cells Using Shotgun Proteomics

**DOI:** 10.3390/medicina61020286

**Published:** 2025-02-07

**Authors:** Hiroko Otake, Tetsushi Yamamoto, Naoki Yamamoto, Yosuke Nakazawa, Yoshiki Miyata, Atsushi Taga, Hiroshi Sasaki, Noriaki Nagai

**Affiliations:** 1 Faculty of Pharmacy, Kindai University, Osaka 577-8502, Japan; 2 Research Promotion Headquarters, Fujita Health University, Toyoake 470-1192, Japan; 3 Faculty of Pharmacy, Keio University, 1-5-30 Shibakoen, Tokyo 105-8512, Japan; 4 Faculty of Pharmacy, Teikyo University, 2-11-1 Kaga, Tokyo 173-8606, Japan; 5 Department of Ophthalmology, Kanazawa Medical University, Uchinada 920-0293, Japan

**Keywords:** shotgun proteomics, lens, temperature, cataract, iHLEC-NY2

## Abstract

*Background and Objectives*: In previous studies, we reported that the assessment of the cumulative thermal dose in the crystalline lens, conducted through computational modeling utilizing a supercomputer and the biothermal transport equation, exhibited a significant association with the incidence of nuclear cataracts. In this study, we have investigated the types of proteins that expressed underlying 35.0 °C (normal-temp) and 37.5 °C (warming-temp) by using the shotgun liquid chromatography (LC) with tandem mass spectrometry (MS/MS)-based global proteomic approach. *Materials and Methods*: We have discussed the changes in protein expression in warmed iHLEC-NY2 cells using Gene Ontology analysis and a label-free semiquantitative method based on spectral counting. *Results*: In iHLEC-NY2, 615 proteins were detected, including 307 (49.9%) present in both lenses cultured at normal-temp and warming-temp, 130 (21.1%) unique to the lens cultured at normal-temp, and 178 (29.0%) unique to the lens cultured at warming-temp. Furthermore, LC–MS/MS analysis showed that warming decreased the expression of actin, alpha cardiac muscle 1, actin-related protein 2, putative tubulin-like protein alpha-4B, ubiquitin carboxyl-terminal hydrolase 17-like protein 1, ubiquitin-ribosomal protein eL40 fusion protein, ribosome biogenesis protein BMS1 homolog, histone H2B type 1-M, and histone H2A.J. in iHLEC-NY2. *Conclusions*: The decreases in the specific protein levels of actin, tubulin, ubiquitin, ribosomes, and histones may be related to cataract development under warming conditions. This investigation could provide a critical framework for understanding the correlation between temperature dynamics and the development of nuclear cataracts.

## 1. Introduction

Cataracts are a leading cause of blindness worldwide. With the increasing lifespan worldwide, the number of individuals whose sight is threatened by this disease is expected to increase. There are four major types of cataracts: cortical, nuclear, posterior subcapsular, and mixed. Different risk factors were associated with each risk type. Epidemiological research has identified multiple factors that are linked to an elevated risk of developing nuclear cataracts (NUCs), including greater sunlight exposure, lower socioeconomic status, poorer nutrition, smoking, cortical cataracts due to diabetes, greater sunlight exposure, and female sex [1,2,3,4]. NUCs have the greatest clinical significance because they are the most common type of cataracts and occur along the visual axis. Treatments that prevent the appearance or delay the progression of NUCs have significant therapeutic value. Previous research has shown that the prevalence of NUCs, graded at level ≥1 according to the World Health Organization (WHO) cataract grading system, was notably higher in tropical and subtropical regions than in temperate and subarctic regions, regardless of racial factors [5,6,7]. Therefore, elevated lens temperatures resulting from higher environmental temperatures may contribute to an increased risk of NUC formation. 

Thus, we hypothesized that the occurrence of cataracts is associated with environmental temperature. The supporting evidence includes a study on ambient temperature effects, where the lens temperature of monkeys exposed to direct sunlight at 49 °C increased to 41 °C within 10 min [8]. Similarly, in rabbits, the lens temperature decreased by 7 °C when maintained in an environment at 4 °C [9]. Another rabbit-based experiment demonstrated significant correlations between ambient temperature under sunlight and the temperatures of the lens and posterior chamber aqueous humor [8].

In this study, we investigated the relationship between environmental temperature and lens temperature through an in silico computer simulation. The lens temperature was estimated to range between 35 °C and 37.5 °C depending on the ambient temperature surrounding the eyeball. However, when the ambient temperature exceeded 30 °C, the estimated lens temperature varied with age, showing an increase in older individuals [10]. Our study showed that, as environmental temperatures rise, the temperature of the eye lens increases to 35–37.5 °C or higher, which correlates with the development of NUCs. The temperature increase, particularly in the lens nucleus, coincides with the opacity area of the cataract. When the lens temperature exceeds 37.5 °C, cumulative heat exposure is positively correlated with NUC incidence [5,10]. This suggests that prolonged exposure to elevated temperatures, especially with aging, may increase the risk of developing NUCs. In addition, we previously investigated the relationship between temperature and NUC incidence in the rat whole lens (including the epithelium, cortex, and nucleus) using a shotgun proteomic analysis approach and showed that the levels of actin, tubulin, vimentin, filensin, and fatty acid-binding protein 5 decreased under warming-temperatures (37.5 °C) [11]. However, it remains unclear whether similar results can be obtained in the human lens, and the detailed mechanisms underlying these findings have yet to be elucidated.

Based on this background, identifying the expression of proteins that fluctuate under warming conditions in human lens cells and discussing preventive measures could contribute to the clinical prevention of NUCs. In this study, we employed a shotgun proteomic analysis approach [12,13] in iHLEC-NY2 (human lens epithelial cells) to investigate the cataractous factors that are relevant to normal and warming conditions.

## 2. Materials and Methods

### 2.1. Culture Cells

The immortalized human lens epithelial cell line iHLEC-NY2 was used as described by Yamamoto et al. [14]. Briefly, the iHLEC-NY2 cell line (source of the cell line “Fujita Health University, Research Promotion Headquarters”), derived from human lens epithelial cells and transfected with modified SV40 large T antigen [15,16], was cultured in medium containing FBS, bFGF, GlutaMAXTM I, DMEM/F12, and penicillin–streptomycin. Cells were cultured at 35.0 °C (normal-temp) and 37.5 °C (warming-temp) in a 5% CO_2_ incubator. The experiment using iHLEC-NY2 was approved by the Ethic Review Committee of Fujita Health University (No. 004, approval date 1 April 2021) and Kanazawa Medical University Biosafety Committee for Recombinant DNA Research (Approval No. 2020-18, approval date 11 November 2020). 

### 2.2. Tryptic Digestion of Proteins Extracted from iHLEC-NY2

iHLEC-NY2 cells were homogenized using at the Minute™ total protein extraction kit for mass spectrometry (Invent Biotechnologies, Inc., Plymouth, MN, USA). Protein concentrations were determined using the Bio-Rad Protein Assay Kit (Bio-Rad Laboratories, Inc., Hercules, CA, USA). Gel-free trypsin digestion was performed as previously described [17]. Briefly, 10 µg of protein extract from each sample was reduced at 37.5 °C for 30 min using 20 mM Tris(2-carboxyethyl)phosphine in 50 mM ammonium bicarbonate buffer and 45 mM dithiothreitol. Subsequently, the proteins were alkylated with 100 mM iodoacetamide in 50 mM ammonium bicarbonate buffer at 37.5 °C for 30 min. Following this alkylation, the samples were digested at 37.5 °C for 24 h using MS-grade trypsin gold (Promega Corp., Madison, WI, USA) at a trypsin-to-protein ratio of 1:100 (*w*:*w*). Finally, the digested peptides were purified using PepClean C-18 Spin Columns (Thermo Fisher Scientific, Waltham, MA, USA) according to the manufacturer’s instructions.

### 2.3. Identification of Proteins

The analysis was performed following our previous study [12,13]. Briefly, peptide samples (2 µg) were injected using a peptide L-trap column (Chemicals Evaluation and Research Institute, Tokyo, Japan) and HTC PAL autosampler (CTC Analytics, Zwingen, Switzerland). Peptide separation occurred on a Paradigm MS4 system (AMR Inc., Tokyo, Japan) with a reverse-phase C18 column (L-column, 3-µm gel particles, 120 Å pore size, and 0.2 mm × 150 mm) at a flow rate of 1 µL/min. The mobile phase consisted of 0.1% formic acid in water (solution A) and acetonitrile (solution B), with gradient elution from 5% to 40% solution B over 120 min. Peptides were analyzed using an LTQ ion-trap mass spectrometer (Thermo Fisher Scientific, Inc.) without sheath or auxiliary gas. MS scan sequences included full-scan MS followed by MS/MS of the two most intense peaks, with parameters optimized for fragmentation. MS/MS data were searched against the SwissProt database using Mascot version 2.4.01, enabling trypsin digestion, missed cleavages, and modifications such as cysteine carbamidomethylation and methionine oxidation. In this study, the fold change in expression was determined as the log2-transformed ratio of protein abundance (*Rsc*) and assessed via spectral counting [18]. *Rsc* was calculated by Equation (1) as follows:

(1) $$Rsc={log}_{2}\frac{n_{s}+f}{n_{n}+f}+{log}_{2}\frac{t_{n}+n_{n}+f}{t_{s}+n_{s}-f}$$

In addition, the normalized spectral abundance factor (*NSAF*) [19] was computed by Equation (2) as follows:

(2) $$NSAF=\frac{{SpC}_{n}/L_{n}}{{SUM(SpC}_{n}/L_{n})}$$

Here, *n_n_* and *n_s_* represent the spectral counts for proteins in rat retinas, whereas *t_n_* and *t_s_* indicate the total spectral counts for all proteins in each sample. The correction factor, denoted as ƒ, was 1.25. *SpC_n_* refers to the spectral count of the protein in rat lenses incubated at normal-temp and warming-temp, while *L_n_* denotes the protein length in these conditions. Proteins were considered differentially expressed when the *Rsc* value was greater than 2 or less than −2, which corresponded to fold changes greater than 2 or less than 0.5, respectively.

### 2.4. Bioinformatics

This study explored the roles of proteins that exhibited notable changes under normal and warming conditions. The sequences were annotated by assigning Gene Ontology (GO) terms corresponding to molecular functions, cellular components, and biological processes, along with Kyoto Encyclopedia of Genes and Genomes (KEGG) signaling pathways, utilizing the Database for Annotation, Visualization, and Integrated Discovery (DAVID, https://davidbioinformatics.nih.gov/tools.jsp, accessed on 3 February 2025) [20,21,22]. Additionally, *p*-values for the GO analysis were computed through this database tool.

## 3. Results

### Protein Expression in iHLEC-NY2 With or Without Warming

**Figure 1:**
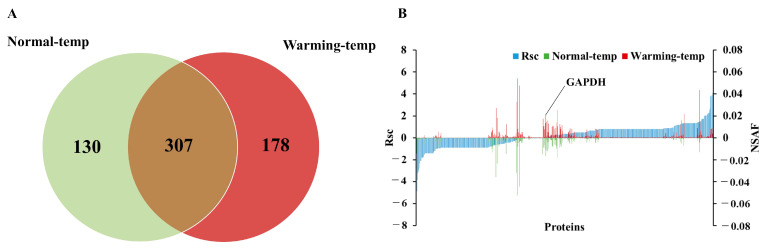
Identification and semiquantitative comparison of the differentially expressed proteins in iHLEC-NY2 cells cultured at normal-temp and warming-temp. (**A**) Venn diagram depicting proteins identified in iHLEC-NY2 cells grown at normal-temp and warming-temp. (**B**) Semiquantitative analysis of proteins differentially expressed in iHLEC-NY2 cells cultured at warming-temp. To compare the expression levels of identified proteins between cells cultured at normal-temp and warming-temp, Rsc and NSAF values were calculated. The blue peak represents Rsc, while the green and red peaks correspond to the NSAF values at normal-temp (lower peak) and warming-temp (upper peak), respectively. Rsc is plotted such that its expression increases from left to right under warming-temp, providing a visual representation of the detected protein behavior. When focusing on GAPDH as a housekeeping protein, it is detected at the approximate center of the x-axis, with NSAF values at normal- (lower peak) and warming-temp (upper peak) showing similar intensities. This consistency suggests that the semiquantitative analysis of proteins was conducted appropriately.

Amounts of 437 and 485 proteins were identified in iHLEC-NY2 cultured at normal-temp and warming-temp, respectively (Figure 1A). Moreover, 615 proteins were detected in iHLEC-NY2, including 307 (49.9%) present in both lenses cultured at normal-temp and warming-temp, 130 (21.1%) unique to the lens cultured at normal-temp, and 178 (29.0%) unique to the lens cultured at warming-temp (Figure 1A). Next, we investigated the proteins expressed in the iHLEC-NY2 cells. Figure 1B shows the Rsc values for the proteins identified in the lenses cultured at normal-temp and warming-temp. A positive Rsc value indicated enhanced expression of proteins in the iHLEC-NY2 cells cultured at elevated temperatures, while a negative value signified reduced expression. Additionally, the NSAF value was computed for each protein identified in iHLEC-NY2 cells cultured at both normal- and warming-temp. Proteins with Rsc values greater than 2 or less than −2 were identified as candidate proteins exhibiting differential regulation in response to the different culture conditions. At different culture temperatures, the housekeeping protein levels (GAPDH, glyceraldehyde-3-phosphate dehydrogenase) did not change. 

We performed a GO analysis on the candidate proteins regulated in the iHLEC-NY2 cells cultured at elevated temperatures. For this analysis, we queried GO terms using the DAVID database, and the results of “molecular function”, “cellular component”, “biological processes”, and “KEGG pathway” are shown in Table 1, Table 2, Table 3 and Table 4, respectively. In the categories of “molecular function”, “cellular component”, “biological processes”, and “KEGG pathway”, the detected counts were 29, 44, 43, and 16, respectively. Among these, the most abundant terms in each category were “protein binding”, “extracellular exosome”, “nucleosome assembly”, and “neutrophil extracellular trap formation”, respectively.

In addition, we listed proteins with expression changes at warming-temp that showed Rsc > 2 or <−2 via the label-free semiquantitative method based on spectral counting (Table 5 and Table 6). The proteins demonstrating Rsc > 2 or <−2 were detected to be 30 in total, and, at warming-temp, the expression levels of 19 proteins were upregulated, while the expression levels of another 11 proteins were downregulated. In this study, our focus was on the downregulated proteins at warming-temp since they are more prone to being influenced than overexpressed proteins. The factors in this study were actin, alpha cardiac muscle 1, actin-related protein 2, putative tubulin-like protein alpha-4B, ubiquitin carboxyl-terminal hydrolase 17-like protein 1, ubiquitin-ribosomal protein eL40 fusion protein, ribosome biogenesis protein BMS1 homolog, histone H2B type 1-M, and histone H2A.J. Keratin was also detected via proteomic analysis. However, because keratin is not present in the lens, the possibility of contamination during lens extraction has been suggested.

## 4. Discussion

Previous research has shown that the prevalence of NUCs, graded at level ≥1 according to the WHO cataract grading system, was notably higher in tropical and subtropical regions than in temperate and subarctic regions regardless of racial factors [5,6,7]. In addition, it was reported that cumulative heat exposure is positively corelated with NUC incidence when the lens temperature exceeds 37.5 °C [5,10]. Thus, elevated lens temperatures resulting from higher environmental temperatures may contribute to an increased risk of NUC formation. However, the exact connection between NUCs and temperature is yet to be fully understood. We demonstrated the types of proteins expressed under normal and warming conditions by using shotgun proteomic analysis and found a decrease in the specific proteins involved in actin, tubulin, ubiquitin, ribosome, and histone under warming conditions in this study.

First, we determined the incubation temperature at normal-temp and warming-temp following a previous computer simulation in silico study [14] and identified 30 proteins exhibiting > 2-fold changes in expression between iHLEC-NY2 under normal-temp and warming-temp. Furthermore, the effect on the expression system is typically more significant when a protein is underexpressed compared to when it is overexpressed. Therefore, we have focused on variations in the expression of 11 factors (the specific proteins concerned were actin, tubulin, ubiquitin, ribosome, and histone), as described in Table 6. Decreased actin and tubulin expression was observed under warming conditions (Table 1). The cytoskeleton of the human eye, comprising actin microfilaments, intermediate filaments, microtubules, and their associated proteins, is essential for cellular growth, maturation, differentiation, integrity, and function. Actin microfilaments are composed of F-actin helices, which are built from G-actin subunits (47 kD) [23,24]. These filaments are distributed throughout the cytoplasm, form a fine mesh under the plasma membrane, or organize into stress fibers. The processes of actin polymerization and depolymerization are modulated by actin-regulatory proteins such as gelsolin. Additionally, various associated proteins bind actin filaments to the plasma membrane, supporting the cellular architecture [23,24]. Therefore, a decrease in actin levels may weaken cell membrane protein binding, resulting in lens opacity. 

The putative tubulin-like protein alpha-4B is a cytoskeletal protein that constitutes a part of a structure known as microtubules. Microtubules play a crucial role in maintaining cell shape, cell division, and intracellular transport. Tubulin forms microtubules by dimerizing α-tubulin and β-tubulin, thereby providing structural stability within cells. The lens cells rely on microtubules to maintain their morphology [25]. Tubulin dysfunction can compromise microtubule stability, thus leading to alterations in cell shape and function, which may result in the loss of lens transparency. Furthermore, microtubules are essential for the proper transport of proteins within cells, including lens cells, where their functions are critical [26]. Abnormalities in putative tubulin-like protein alpha-4B may disrupt protein transport, potentially causing protein aggregation within the lens. This aggregation contributes to lens opacification. Additionally, because microtubules are involved in the proliferation and maintenance of lens cells, tubulin defects can lead to cellular dysfunction, which may contribute to lens opacity. Therefore, the putative tubulin-like protein alpha-4B plays a vital role in maintaining the structural integrity of lens cells and protein transport. A reduction in putative tubulin-like protein alpha-4B under high-temperature conditions may be one of the factors that contribute to lens opacification. 

In addition, the expression of the proteins related to ubiquitin and ribosome in warming-temp-incubated iHLEC-NY2 was also lower than that in normal-temp-incubated iHLEC-NY2. Many of the signals that maintain lens epithelia appear to be substrates of the ubiquitin–proteasome pathway [27]. Ubiquitin C-terminal hydrolase L17-like protein 1 is an enzyme that is responsible for protein degradation and is particularly involved in the ubiquitin–proteasome system, a key protein quality control mechanism [28,29]. This system is essential for preserving cellular homeostasis by facilitating the elimination of damaged or misfolded proteins.

The eL40 fusion protein consists of ubiquitin, which tags damaged or unnecessary proteins for degradation, and the ribosomal protein eL40, which is involved in protein synthesis [30]. The BMS1 homolog is crucial for ribosome assembly, particularly ribosomal RNA (rRNA) processing and ribosomal subunit assembly [31]. Ribosomes are essential for protein synthesis within cells, and proteins such as BMS1 are indispensable for the proper formation of functional ribosomes [32]. Impairment of ribosome biogenesis can lead to increased production of misfolded proteins, especially in long-lived cells such as lens cells, which can contribute to cataract formation. Therefore, dysfunctions or mutations in BMS1 may increase the risk of cataract development.

Histones are key proteins involved in DNA packaging within the nuclei of eukaryotic cells. They wrap DNA to form chromatin, thus enabling it to be compactly stored and to regulate gene expression [33]. If histone modifications or structural changes adversely affect the expression of genes that are critical for maintaining lens transparency, improper protein folding and aggregation within the lens may occur, leading to loss of lens transparency.

Moreover, we examined their functions by analyzing the four GO terms (Table 1, Table 2, Table 3 and Table 4). The GO analysis indicated that the most common factors identified in the molecular function, cellular component, biological processes, and KEGG pathway categories were “protein binding”, “extracellular exosome”, “nucleosome assembly”, and “neutrophil extracellular trap formation”, respectively (Table 1, Table 2, Table 3 and Table 4). The proteins involved in protein binding were actin and alpha cardiac muscle 1. The proteins associated with extracellular exosomes included actin, alpha cardiac muscle 1, actin-related protein 2, ribosome biogenesis protein BMS1 homolog, histone H2B type 1-M, and histone H2B type 1-M. Additionally, the protein involved in nucleosome assembly was histone H2B type 1-M. Taken together, it is possible that factors associated with actin, ribosomes, and histones are specifically involved in the onset of cataracts due to temperature changes. However, the present results also show that the expression of other proteins related to tubulin and histones, such as tubulin alpha-1C chain and histone-H2B type 1-C/E/F/G/I, -H2B type F-S, -H2B type 1-D, -H2A type 1-H, and -H3.1, increases at warming-temp (Table 5). Therefore, changes in the tubulin and histone levels may be associated with homeostatic maintenance. Further investigations are required in order to determine whether the decrease or increase in these proteins at higher ambient temperatures plays a dominant role.

It is crucial to explore whether the overexpression of certain proteins and the reduction in others at elevated temperatures are associated with lens dysfunction. In our previous study utilizing a similar shotgun proteomic analysis, we demonstrated that heating the rat whole lens (including the epithelium, cortex, and nucleus) at warming-temp resulted in reductions in actin, tubulin, vimentin, filensin, and fatty acid-binding protein 5 [11]. Among these, both actin and tubulin were found to decrease upon heating in both the rat lens and iHLEC-NY2. These findings suggest that the observed reductions in actin and tubulin may at least be attributable to epithelial cells. Thus, this study has successfully screened lens proteins that change in response to elevated temperature, which were previously unidentified as potential causes of NUCs. As a result, it is now possible to consider temperature-related factors in NUC development, contributing to future research advancements. However, this study does not fully reflect the changes occurring in the nuclear or cortical regions of the lens since human epithelial cells were used. Moreover, additional research is required to assess the relationship between the onset of NUCs and changes in the proteins involved in actin, tubulin, ubiquitin, ribosomes, and histones. Therefore, we are planning to measure the localization and expression of the specific proteins concerning actin, tubulin, ubiquitin, ribosomes, and histones under warming-temp by using Western blotting and an immunostaining method.

## 5. Conclusions

The conducted shotgun proteomic analysis revealed that warming decreased the expression of specific proteins involved in actin, tubulin, ubiquitin, ribosomes, and histones in iHLEC-NY2. This study could provide a valuable framework for understanding the relationship between temperature and the onset of NUCs. However, additional research is necessary to fully comprehend the mechanisms that link these factors. In addition, regarding the clinical correlation of shotgun proteomics and future directions, it is desirable to investigate whether similar protein fluctuations occur using postoperative samples from human NUC patients. Furthermore, establishing prevention or treatment strategies for nuclear cataracts by suppressing these protein fluctuations is anticipated.

## Figures and Tables

**Table 1:** GO analysis of identified proteins in molecular function category.

Molecular Function Category	Relative Abundance (%)	Molecular Function Category	Relative Abundance (%)
Protein binding	77.1	Unfolded protein binding	6.37
RNA binding	32.5	Structural molecule activity	5.73
DNA binding	29.3	Structural constituent of muscle	4.46
Protein heterodimerization activity	28.0	Double-stranded DNA binding	3.18
Structural constituent of chromatin	27.4	Protein binding involved in protein folding	3.18
ATP binding	15.9	Collagen binding	2.55
Protein domain specific binding	12.1	Heat shock protein binding	2.55
Cadherin binding	11.5	Microfilament motor activity	2.55
GTP binding	8.28	Misfolded protein binding	2.55
Structural constituent of cytoskeleton	8.28	Motor activity	2.55
ATPase activity	7.64	Structural constituent of epidermis	2.55
GTPase activity	7.64	mRNA 5’-UTR binding	1.91
Structural constituent of ribosome	7.64	Protein disulfide isomerase activity	1.91
Actin binding	6.37	Large ribosomal subunit rRNA binding	1.27
Actin filament binding	6.37		

**Table 2:** GO analysis of identified proteins in cellular component category.

Cellular Component Category	Relative Abundance (%)	Cellular Component Category	Relative Abundance (%)
Extracellular exosome	63.7	Cytosolic large ribosomal subunit	4.46
Nucleus	58.6	Ribonucleoprotein complex	4.46
Cytosol	53.5	Cytosolic small ribosomal subunit	3.82
Cytoplasm	41.4	Ficolin-1-rich granule lumen	3.82
Nucleoplasm	39.5	Actin filament	3.18
Membrane	36.9	Meiotic spindle	3.18
Nucleosome	28.0	Small-subunit processome	3.18
Extracellular region	22.9	Vesicle	3.18
Macromolecular complex	21.7	Z disc	3.18
Focal adhesion	15.3	Intercellular bridge	2.55
Nuclear chromosome	15.3	Myosin complex	2.55
Extracellular space	14.7	Myosin II complex	2.55
Endoplasmic reticulum	12.7	Ruffle membrane	2.55
CENP-A-containing nucleosome	10.2	Sarcomere	2.55
Chromosome, telomeric region	10.2	Small ribosomal subunit	2.55
Perinuclear region of cytoplasm	8.28	Smooth endoplasmic reticulum	2.55
Ribosome	8.28	Endoplasmic reticulum chaperone complex	1.91
Cytosolic ribosome	7.01	Myosin filament	1.91
Melanosome	5.73	Polysome	1.91
Endoplasmic reticulum lumen	5.10	CRD-mediated mRNA stability complex	1.27
Intermediate filament	5.10	Muscle thin filament tropomyosin	1.27
Microtubule	5.10	Myosin II filament	1.27

**Table 3:** GO analysis of identified proteins in biological process category.

Cellular Component Category	Relative Abundance (%)	Cellular Component Category	Relative Abundance (%)
Nucleosome assembly	23.6	Cytoskeleton organization	3.82
Chromatin organization	17.2	Microtubule-based process	3.82
DNA replication-dependent nucleosome assembly	15.3	Actomyosin structure organization	3.18
Telomere organization	15.3	Muscle contraction	3.18
Protein localization to CENP-A containing chromatin	10.2	Oocyte maturation	3.18
DNA-templated transcription, initiation	9.55	Osteoblast differentiation	3.18
DNA replication-independent nucleosome assembly	8.92	Ribosomal small subunit biogenesis	3.18
Negative regulation of megakaryocyte differentiation	8.92	Spindle assembly involved in female meiosis	3.18
Cytoplasmic translation	7.64	Cellular response to unfolded protein	2.55
Negative regulation of apoptotic process	7.64	Chaperone mediated protein folding requiring cofactor	2.55
Translation	7.64	Protein folding in endoplasmic reticulum	2.55
Gene expression	7.01	Protein refolding	2.55
Regulation of gene expression, epigenetic	6.37	Actin filament-based movement	2.55
Protein folding	5.73	Cellular copper ion homeostasis	1.91
Antibacterial humoral response	5.10	Cellular response to interleukin-7	1.91
Antimicrobial humoral immune response mediated by antimicrobial peptide	5.10	Dendritic spine organization	1.91
Defense response to Gram-positive bacterium	5.10	Long-term synaptic depression	1.91
Heterochromatin assembly	5.10	Mitotic cleavage furrow ingression	1.91
Innate immune response in mucosa	5.10	Regulation of Arp2/3 complex-mediated actin nucleation	1.91
Mitotic cell cycle	5.10	Regulation of receptor internalization	1.91
Intermediate filament organization	4.46	Skeletal muscle myosin thick filament assembly	1.27
Microtubule cytoskeleton organization	4.46		

**Table 4:** GO analysis of identified proteins in pathway category.

Molecular Function Category	Relative Abundance (%)	Molecular Function Category	Relative Abundance (%)
Neutrophil extracellular trap formation	26.7	Protein processing in endoplasmic reticulum	6.37
Alcoholism	25.5	Motor proteins	5.73
Systemic lupus erythematosus	25.5	Parkinson disease	5.73
Viral carcinogenesis	17.2	Prion disease	5.73
Shigellosis	9.55	Pathogenic Escherichia coli infection	5.10
Coronavirus disease—COVID-19	7.01	Necroptosis	4.46
Ribosome	7.01	Estrogen signaling pathway	3.82
Transcriptional misregulation in cancer	7.01	Antigen processing and presentation	3.18

**Table 5:** Semiquantitative comparison of proteins with increased expression in iHLEC-NY2 cultured under warming-temp conditions.

ID	Accession Number and Description		Number of Amino Acids	Spectral Counting		
				Warming-Temp	Normal-Temp	Fold Change, Rsc
TBA1C_HUMAN	Q9BQE3	Tubulin alpha-1C chain	449	89	0	6.156903
HS902_HUMAN	Q14568	Heat shock protein HSP 90-alpha	343	21	0	4.122102
H2B1C_HUMAN	P62807	Histone H2B type 1-C/E/F/G/I	126	18	0	3.912511
H2BFS_HUMAN	P57053	Histone H2B type F-S	126	17	0	3.834335
H2B1D_HUMAN	P58876	Histone H2B type 1-D	126	17	0	3.834335
H2A1H_HUMAN	Q96KK5	Histone H2A type 1-H	128	16	0	3.753820
H31_HUMAN	P68431	Histone H3.1	136	7	0	2.687759
RS3A_HUMAN	P61247	Small ribosomal subunit protein eS1	264	6	0	2.501132
PGAM2_HUMAN	P15259	Phosphoglycerate mutase 2	253	5	0	2.286793
RLA0L_HUMAN	Q8NHW5	Putative ribosomal protein uL10-like	317	5	0	2.286793
TGM2_HUMAN	P21980	Protein-glutamine gamma-glutamyltransferase 2	687	19	3	2.219596
HS71L_HUMAN	P34931	Heat shock 70 kDa protein 1-like	641	14	2	2.196653
HSP76_HUMAN	P17066	Heat shock 70 kDa protein 6	643	18	3	2.146318
CNGB1_HUMAN	Q14028	Cyclic nucleotide-gated cation channel beta-1	1251	4	0	2.035040
RPN1_HUMAN	P04843	Dolichyl-diphosphooligosaccharide--protein glycosyltransferase subunit 1	607	4	0	2.035040
RL15_HUMAN	P61313	Large ribosomal subunit protein eL15	204	4	0	2.035040
MYH14_HUMAN	Q7Z406	Myosin-14	1995	4	0	2.035040
TAF9B_HUMAN	Q9HBM6	Transcription initiation factor TFIID subunit 9B	251	4	0	2.035040
PDIA4_HUMAN	P13667	Protein disulfide-isomerase A4	645	8	1	2.004817

**Table 6:** Semiquantitative comparison of proteins with decreased expression in iHLEC-NY2 cultured under warming-temp conditions.

ID	Accession Number and Description		Number of Amino Acids	Spectral Counting		
				Warming-Temp	Normal-Temp	Fold Change, Rsc
ACTC_HUMAN	P68032	Actin, alpha cardiac muscle 1	377	0	81	6.094116
H2B1M_HUMAN	Q99879	Histone H2B type 1-M	126	0	34	4.861313
K2C75_HUMAN	O95678	Keratin, type II cytoskeletal 75	551	0	16	3.826320
H2AJ_HUMAN	Q9BTM1	Histone H2A.J	129	0	10	3.208328
TBA4B_HUMAN	Q9H853	Putative tubulin-like protein alpha-4B	241	0	9	3.073807
K1C26_HUMAN	Q7Z3Y9	Keratin, type I cytoskeletal 26	468	0	8	2.925489
U17L1_HUMAN	Q7RTZ2	Ubiquitin carboxyl-terminal hydrolase 17-like protein 1	530	0	7	2.760210
K2C79_HUMAN	Q5XKE5	Keratin, type II cytoskeletal 79	535	0	5	2.359232
RL40_HUMAN	P62987	Ubiquitin-ribosomal protein eL40 fusion protein	128	0	4	2.107474
ARP2_HUMAN	P61160	Actin-related protein 2	394	0	4	2.107474
BMS1_HUMAN	Q14692	Ribosome biogenesis protein BMS1 homolog	1282	0	4	2.107474

## Data Availability

The raw MS data files were deposited in the ProteomeXchange Consortium via the jPOST partner repository (http://jpostdb.org, accessed on 18 January 2025) under the dataset identifier PXD059029/JPST003507. Publicly available datasets, such as the UniProt dataset (https://www.uniprot.org/help/downloads, accessed on 18 January 2025) utilized in this study, can also be accessed through their respective repositories following the guidelines provided by the data-sharing platforms. The data generated in this study can be requested from the corresponding author.

## Abbreviations

DAVID	Database for Annotation, Visualization, and Integrated Discovery
GO	Gene Ontology
KEGG	Kyoto Encyclopedia of Genes and Genomes
LC–MS/MS	Liquid chromatography with tandem mass spectrometry
NSAF	Normalized spectral abundance factor
NSI	Nanoelectrospray ionization
NUC	Nuclear cataract
Rsc	log2-transformed ratio of protein abundances
